# EFL teachers’ digital literacy: the role of contextual factors in their literacy development

**DOI:** 10.3389/fpsyg.2023.1153339

**Published:** 2023-07-24

**Authors:** Jie Zhang

**Affiliations:** Faculty of Foreign Languages and Business, Jiaozuo Normal College, Jiaozuo, China

**Keywords:** digital literacy, technology-based teaching, teachers’ beliefs, teaching experience, EFL

## Abstract

**Introduction:**

Digital technology can have significant effects on language education. This effect makes the English language teachers teach the subjects better to the students and also improves the quality of teachers’ education.

**Methods:**

The purpose of this research is to evaluate the digital literacy of English language teachers and to investigate the difference between digital literacy and their gender, education level, and teaching experience. To conduct the study, the researcher invited 2,110 EFL teachers to fill out the Teachers’ Digital Literacy questionnaire. The researchers used SPPS and AMOS in analyzing the obtained data.

**Results:**

The results of the study indicated that teachers’ contextual factors do not influence their digital literacy skills. In addition, the study showed that teachers’ attitudes toward technology, their skill to use technology, and their access to technology can significantly affect teachers’ digital literacy.

**Discussion:**

Implications of the study are further discussed in this paper.

## Introduction

Digital literacy can influence teachers’ performances and developments. Information and communication technologies have been effective in every aspect of life, and as a result, many changes have occurred in different ways of doing our regular work (Gao et al., 2022; Wang, 2023; Wang Y. L. et al., 2023; Wang et al., 2023a). It is necessary for English teachers to be equipped with these skills to prepare for future jobs. The position of digital literacy in teaching and learning has not been emphasized as much as it should be. In line with the professionalization of English language teachers, increasing their literacy seems necessary and vital (Belda-Medina, 2022). The traditional concept of literacy refers to the ability to read and write. In the 1990s, the emergence of digital technology changed literacy based on paper, tradition, and knowledge into modern and social computer literacy (Carolus et al., 2023). In other words, literacy was redefined in this period as a collaborative concept in different times and places. This literacy through multimedia resources can lead to creating favorite materials, changing the shape of language learning, increasing critical thinking, and sharing views with others (Chik, 2011; Guo et al., 2023).

The emergence of digital technology brought different types of literacy, including media literacy (Mellati et al., 2015; Fernández-Batanero et al., 2022; Fu and Wang, 2022; Wang et al., 2023b), computer literacy (Gruszczynska and Pountney, 2013; Wang, 2023), information and internet literacy (Belda-Medina, 2022), and digital literacy (Carolus et al., 2023). Digital literacy can include all the mentioned literacy. Digital technology created new opportunities for students and teachers to improve their skills and access valid materials using the digital space (Hutchison and Woodward, 2018). Also, this technology can bring the creativity of teachers and language learners. Digital tools make classrooms more dynamic, cooperative, attractive, and valuable (Jeong, 2017). It also increases the creativity and critical thinking of both language learners and teachers, and as a result, it leads to their greater independence. Teachers will be able to release students from the consumer mode and turn them into active and creative students by using different types and forms of multimedia. In addition, digital tools improve the two-way communication between teachers and learners and facilitate teaching and learning process (List, 2019; Mellati and Khademi, 2020).

However, digital technology has its limitations. For instance, digital spaces may be used more for entertainment and less for educational purposes (Murphy and Lebans, 2009). For instance, Palacios-Hidalgo and Huertas-Abril (2022) argued that a lack of digital literacy could expose teachers and students to inappropriate and invalid content and distance them from the real world or lead to addiction to technology. In addition, Bond et al. (2019) argued that this crucial issue would lead to the risk of the digital divide in terms of access to digital facilities and the ability to use them (Tamborg et al., 2018; Mellati and Khademi, 2019; Belda-Medina, 2022). However, the advantages of digital technology are more than its disadvantages. In other words, digital technology skills can lead to the professional development and empowerment of teachers, improve the quality of their education, and self-confidence and mastery in using these technologies (Pérez-Escoda et al., 2019).

Despite the new and different types of digital technology, the movement from writing and speaking to digital style has led to essential changes in how teachers teach (Mellati et al., 2018; Shively and Palilonis, 2018). Language teachers should have the right and appropriate choice of digital technology for effective and efficient training; therefore, they need to acquire digital literacy (Zhao et al., 2018; Fu and Wang, 2022). The lack of this literacy can hinder the optimal and appropriate use of digital technology. As a result, research and study of digital literacy among English language teachers is necessary and important (Zimmer et al., 2021). Acquiring dominance over digital technology includes the capacity to get access to, produce, and offer digital data, which has been simpler than before because of the utilization of the internet. To be sure, the internet sets out open doors for being adaptable with regards to sharing or recovering data. Moreover, teachers ought to be outfitted with the basic and logical abilities, which are expected for handling data acquired through the Internet to build up learning. Thusly, digital literacy emerges as it has a critical capability in language training like never before. Teacher digital literacy is seen as a fundamental component of training that considers the viable utilization of Information Communication Technology (ICT). This sort of literacy has influenced the nature of instructing, consequently assuming a developing critical part in training. It is occupant on all teachers to draw on their ICT abilities while instructing (Zhao et al., 2018).

Many factors affect the digital literacy of teachers, which can be mentioned as a willingness. They will have optimistic view of technology, knowledge of using technology, access to technology, and contextual factors. However, gender, teaching experience, and educational level are factors affecting the digital literacy of teachers. Reviewing the literature reveals that few studies had been conducted in evaluating the difference between digital literacy and contextual factors such as gender, education level, and teaching experience of English language teachers. As a result, the current research aim is to fill this research gap by proposing the following three questions:

## Research questions

*RQ1*. What are Chinese EFL teachers’ perceptions of digital literacy?

*RQ2*. Is there any significant association between EFL teachers’ demographic factors (age, gender, educational level, and teaching experience) and their differences in their technology literacy, their digital participation, engagement, and perceptions of technology integration?

*RQ3*. Is there a significant association between EFL teachers’ demographic factors (age, gender, educational level and teaching experience), technology literacy, and their perceptions of the barriers to technology integration (motivation, technology literacy, and technology access)?

## Review of the literature

In this section, digital literacy is discussed from different perspectives. First, the definitions of digital literacy are discussed. Then its importance is examined. Finally, the studies that have been done in this field in different environments are discussed.

### Digital literacy

Digital literacy is the skills you need to live, learn, and work in a society where communication and access to information are through digital technologies like internet platforms, social media, and mobile devices (Hutchinson and Novotny, 2018). Education is the only way to achieve balanced development and scientific progress. The most important concern of the country’s education system is to create a suitable platform for the growth and flourishing of intellectual capital in a knowledge-oriented society (Jensen, 2019). The surveys conducted regarding the amount of knowledge, ability, and educational attitudes of teachers indicate the fact that comprehensive mastery of teachers in the academic and specialized fields of their profession is not provided to the desired extent (Craig, 2013; Meesong and Jaroongkhongdach, 2016; Hallinger and Hosseingholizadeh, 2020; Pikhart et al., 2022). The development of teachers’ discourse in its current form has had minimal impact on teaching methods, curricula and what students should learn. Rapid changes, pervasive use of technology, and increasing access to information volume have necessitated professional development. Therefore, it is necessary to establish a relationship between education and the professional development of teachers and to strengthen their motivation to learn (Gilmore, 2012; Richards, 2017; Mellati and Khademi, 2019). Professional development of teachers causes changes in teaching methods and improves students’ learning results. Professional development of teachers through authentic structures is considered for profound teaching and learning (Beikmohammadi et al., 2020).

In addition, the role of technology in the impact of language cannot be ignored. Belda-Medina (2022) believes that digital technology has deeply affected language and its usage in people’s daily life. Digital media have become so intertwined with the lives of ordinary people that nowadays, our correspondence is done through these media rather than face-to-face. From Bond et al. (2019) point of view, these effects have appeared in different aspects. Two important ones can be mentioned. The first effect is the abundance of new words. The second substantial effect is our use of language in online and virtual environments. The way we read, write, communicate with others, apply concepts, share our knowledge with others, and finally, our understanding of ourselves concerning the world around us is affected by this emerging phenomenon (Chik, 2011; Centre et al., 2012; Carolus et al., 2023). Therefore, according to Fernández-Batanero et al. (2022), literacy, which is considered a mediating element between language and technology while maintaining its traditional meaning, has become a dynamic phenomenon under this new technology, which can be interpreted as digital literacy.

Researchers have given several definitions of digital literacy. Gilster and Glister (1997) were the first to propose digital literacy. They considered it a set of capabilities and abilities of students to use the Internet only to obtain official information in the framework of the school. With the passage of two decades, digital literacy, with its change, expansion, and evolution, also included other concepts such as image literacy (George and Sanders, 2017), reproduction literacy (Gisbert Cervera and Lázaro Cantabrana, 2015), information literacy (Gruszczynska and Pountney, 2013) and social–emotional literacy (Gudmundsdottir and Hatlevik, 2018). Digital literacy is not necessarily the acquisition of a set of skills and abilities to manage online data. This important thing can help people identify, access, manage, share, evaluate (Alavi et al., 2022), combine digital resources, produce science, create new media terms, and establish relationships with others (Hutchison, 2012; Hall et al., 2014; Ilomäki et al., 2016; Gurevich et al., 2017; Hutchison and Woodward, 2018). Based on this point of view, digital literacy goes beyond acquiring the basic skills of information and communication technology for the application of technology, including the awareness, attitude, ability of people to identify, access, manage, complete, evaluate, analyze, and combine content and digital resources, create new knowledge, create Media expressions, and communication with others are in the context of special life conditions (Ilomäki et al., 2016; Jeong, 2017; Pettersson, 2018; List, 2019; Palacios-Hidalgo and Huertas-Abril, 2022; Tawafak et al., 2023).

Having digital literacy does not necessarily mean knowledge that can solve problems (Instefjord and Munthe, 2017). The first fundamental problem related to digital literacy is individualism, cultural tradition, and human identities, which can lead to local and ethnic views in the monopoly of English-speaking countries (Jeong, 2017). The second primary issue in this field is emphasizing the role of technology without considering environmental and contextual factors (Katia and José Tejada, 2018). With all the existing problems, digital literacy through digital technology can be developed globally and play an important and prominent role in educational, employment, and evaluation fields (List, 2019). Next, the importance and necessity of digital literacy for language teachers are discussed.

### The importance of digital literacy of teachers

One of the main goals of education is to create correct and appropriate classroom changes (Murphy and Lebans, 2009). Naturally, one of these factors can be the digital literacy of teachers (Palacios-Hidalgo and Huertas-Abril, 2022). In order to improve this literacy, teachers should respond positively to modern and inevitable changes and adapt themselves to these changes (Pérez-Escoda et al., 2019). They should change their traditional way of teaching, thinking, and perspective to improve their teaching process in the classrooms (Pettersson, 2018).

Many studies have confirmed this fact (Gurevich et al., 2017; Jeong, 2017; Hutchison and Woodward, 2018; Zimmer et al., 2021; Palacios-Hidalgo and Huertas-Abril, 2022; Carolus et al., 2023). For example, Pérez-Escoda et al. (2019) argued that teachers’ identity links to digital tools, the correct use of technology, the expression of personal views, and their understanding of their skills and self-confidence in digital environments. Another factor in improving teachers’ digital literacy is access to digital facilities and resources. Access to technology can increase innovation in teaching and learning methods (Instefjord and Munthe, 2017). Based on List’s (2019) research, teachers were able to improve their digital skills, develop their creativity and problem-solving skills, and become independent in their learning and teaching by using various digital technologies. Considering the importance of digital literacy, teachers can help uninterested and hesitant students to take the initiative and cooperate in classroom activities. They can give them cognitive and metacognitive independence and awareness (Murphy and Lebans, 2009). In addition, using digital tools facilitates and strengthens the remote participation capabilities of teachers (Bond et al., 2019). Teachers equipped with digital literacy can pave the way for learners to experience the joy of freedom of action, encouragement, motivation, and success in personal and academic life (Gudmundsdottir and Hatlevik, 2018).

### A framework for emergent digital literacy

**Figure 1:**
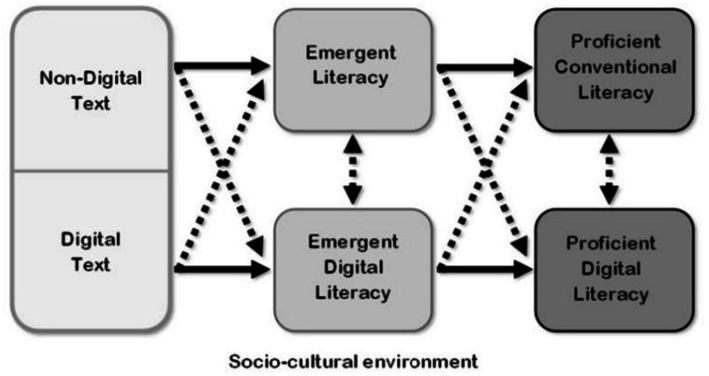
Digital literacy framework (Neumann et al., 2017).

To supplement new education abilities, a comprehensive framework for mechanical proficiency improvement is proposed. This is displayed in Figure 1. As currently noted, sociocultural communications with non-advanced and computerized texts will advance the progress of rising proficiency and developing computerized education abilities. The spotted line encompassing the framework features the significance of surveying this model inside a dynamic socio-social climate where guardians, vocations, educators, peers, and developing social apparatuses (e.g., digital books, computerized games, applications, and mechanical technology coding programming) assume a crucial part in interceding youngsters’ encounters with text (Neumann et al., 2017).

### Studies conducted about digital literacy

Attitude, access, and skill in using digital resources are influential factors that cover teachers’ digital literacy (Centre et al., 2012). The results of recent studies have shown that digital literacy has a stimulating role in using digital tools among teachers (Hall et al., 2014; Gurevich et al., 2017; Gudmundsdottir and Hatlevik, 2018). In a similar research, Shively and Palilonis (2018) pointed out that the authorities should pay more attention to the role of digital technology in schools and prepare the access and use of it for teachers and students in the direction of their expected success. Other studies have emphasized the benefits of digital literacy (Hall et al., 2014; Instefjord and Munthe, 2017; List, 2019; Mellati et al., 2022; Palacios-Hidalgo and Huertas-Abril, 2022).

For example, Spiteri and Chang Rundgren (2017) studied life changes based on digital literacy. They found that this type of literacy, despite its limitations, can cultivate capable and creative people in society. Also, with a combined study on in-service teachers, Tondeur et al. (2017) found that direct and explicit teaching of digital literacy through electronic books can increase the level of digital literacy of students in technical, cognitive, and emotional–social fields, as a result, reduce their cognitive load. In other words, students can concentrate easily on their content and tasks. This study indicated that teachers familiar with digital literacy have more ability to manage unfamiliar digital spaces, as a result, can create their digital literacy profile and share it with others in virtual learning environments.

Some other researchers have studied the digital literacy exercises of the students and their level of progress (Gurevich et al., 2017; Gudmundsdottir and Hatlevik, 2018; Hutchison and Woodward, 2018). The results of Zhao et al. (2018) qualitative study showed that the students were more involved with the four dimensions of digital literacy, which can be mentioned as basic computer use, information search, thoughtful use of digital technology, and multimedia content production. Zimmer et al. (2021) study concerning students’ digital exercises showed that students could use their skills in learning aspects such as writing exercises and thus change the perspective of education about the practical construction of these activities in curricula. They concluded that digital environments can transform students’ exercises and homework and help officials improve their understanding of digital tools in their personal and academic lives.

The relationship between identity and digital literacy has also been studied. Gudmundsdottir and Hatlevik (2018), in multilingual activities, a student with his multilingual training in the digital space was able to show a multilingual identity, increase his creativity, and communicate with others. In a similar case, List (2019) indicated that digital literacy could lead to forming a new identity by preserving cultural, linguistic, political, and glacial principles. Pérez-Escoda et al. (2019), by studying the teachers during the service, showed that according to the appropriate and correct use of technology, the expression of the students’ point of view and their understanding of their self-confidence and digital skills, can be concluded that the identity of the teachers is related to their digital activities and exercises.

Although researchers have examined emotional literacy, evaluative literacy, and other literacy, most of the studies have been on the use of technology in teaching and learning. For instance, Belda-Medina (2022) evaluated the use of information and communication technology among English language teachers. He found that teachers are more interested in using portable technology (mobile, tablet, and laptop) to teach oral skills. In another study, Fernández-Batanero et al. (2022) investigated computer literacy and multimedia literacy of English language teachers in language schools. The results of the data analysis showed that the computer literacy of English language teachers was relatively high. Still, their information literacy and multimedia literacy were low to average, and they did not have the necessary abilities and skills in these two literacies. Therefore, they concluded that English language teachers should have a good knowledge of technology in order to be able to implement new technology tools practically in the classrooms.

The review of previous studies showed that although the role of digital technology, including using computers for teaching and learning, has been investigated, these studies have not investigated digital literacy, the use of digital technology, and environmental factors. Therefore, the present study aims to evaluate the level of digital literacy of English language teachers and the effect of individual characteristics such as gender, education level, and teaching experience on their digital literacy.

## Methods

### Participants

Two thousand one hundred and ten EFL teachers participated in this survey research. Five hundred thirty-seven of the participants were male, making up 25.45 percent, and 1,573 were female, making up 74.55 percent. Their majors were English Language Teaching, English Translation, and English Literature. There are 1,315 participants majoring in English Language Teaching, making up 62.32 percent; 371 majors in English Translation who make up 10.57 percent, and 424 majors in English Literature make up 20.09 percent. Since the researchers tried to collect authoritative data, they distributed the questionnaire in any possible province. Through convenience sampling, the researchers distributed the questionnaire as much as possible. They taught English in 24 regions that were Beijing, Shaanxi, Guangdong, Guizhou, Anhui, Jiangsu, Gansu, Shandong, Hebei, Xinjiang, Fujian, Shanghai, Hubei, Sichuan, Zhejiang, Shanxi, Qinghai, Yunnan, Chongqing, Hunan, Heilongjiang, Ningxia, Tianjin, Jiangxi, Henan. There are 45 participants from Beijing, accounting for 2.132%; 11 participants from Guangdong who account for 0.521%; 10 participants from Guizhou who account for 0.474%; 14 participants from Anhui who account for 0.664%; 15 participants from Jiangsu who account for 0.711%; 25 participants from Gansu who account for 1.184%; 11 participants from Shandong who account for 0.521%; 5 participants from Hebei who account for 0.237%; 5 participants from Xinjiang who account for 0.237%; 3 participants from Fujian who account for 0.142%; 9 participants from Shanghai who accounting for 0.426%; 10 participants from Hubei who account for 0.474%; 7 participants from Sichuan who account for 0.332%; 30 participants from Zhejiang who account for 1.422%; 12 participants from Shanxi who account for 0.142%; 3 participants from Shaanxi who account for 0.474%; 10 participants from Qinghai who accounting for 0.426%; 9 participants from Yunnan who accounting for 0.043%; 2 participants from Chongqing who accounting for 0.095%; 5 participants from Hunan who accounting for 0.237%; 1 participants from Heilongjiang who account for 0.758%; 16 participants from Ningxia who account for 0.014%; 2 participants from Tianjin who accounting for 0.095%; 4 participants from Jiangxi who accounting for 0.2%; 1851 participants from Henan who account for 87.725%. The study adhered to primary research ethics. The participants had been informed that the information provided would be kept confidential and used only for research purposes. There was no prior association between the researcher, the participants, and no conflict of interest. The authenticity and reliability of the data collected has been checked carefully.

### Instruments

#### Digital literacy questionnaire

The Digital Literacy Questionnaire was the only instrument that was employed in this study. The first version of the questionnaire was developed from the answers of three experts in the field to the interview questions. The first version of the questionnaire had 101 questions. Three experts checked its face and content validity and delivered their comments. Based on the provided comments, the researchers revised the questionnaire. The revised version was subjected to exploratory and confirmatory factor analysis. The final version of the questionnaire with 52 questions was piloted by 50 participants from the same population. The results show a reliability of 0.8 (*r* = 0.80). This questionnaire has six sections; the first section specifies the ethnographic information of the participants, the second section specifies the participants’ attitudes toward technology and their skill in conducting it in their classrooms, the third section specifies their participation in technology-based learning environments, the forth section specifies their engagement with technology in their learning contexts, the fifth section specifies their perceptions of technology use in their learning environments. The last section determines their attitudes toward the barrier to use technology in the language classroom and enhance teachers’ and learners’ digital literacy. This questionnaire measures four key concepts. The more items we include in the analysis, the higher Cronbach’s α will be, but such a higher alpha may not be able to reflect the real internal consistency of an instrument. To control this issue, the researchers reported the α of different constructs of the questionnaire, respectively, rather than regarding them as a whole. The results of the Cronbach Alpha Coefficient show a reliability of 0.75 (*r* = 0.75) for digital participation, 0.78 (*r* = 0.78) for attitudes, 0.83 (*r* = 0.83) for engagement, and 0.79 (*r* = 0.79) for perceptions.

### Procedure

The participants had been informed that the information provided would be kept confidential and used only for research purposes. There was no prior association between the researcher and the participants, and there was no any conflict of interest. The data collected has been carefully checked to ensure that it is authentic and reliable. The researchers used convenience sampling to collect the required data. They distributed the questionnaire link on social networks (WeChat). The researchers distributed the questionnaire among 2,500 EFL teachers. In this survey research, from 2,500 distributed questionnaires, 2,110 questionnaires were received. The overall unweighted response rate for this survey was 84 percent. Ensure about the understanding of the items, the researchers used the questionnaire in English and Chinese. The data collection procedure lasted six working days. The data was collected from 24 provinces of China that were Beijing, Shaanxi, Guangdong, Guizhou, Anhui, Jiangsu, Gansu, Shandong, Hebei, Xinjiang, Fujian, Shanghai, Hubei, Sichuan, Zhejiang, Shanxi, Qinghai, Yunnan, Chongqing, Hunan, Heilongjiang, Ningxia, Tianjin, Jiangxi, Henan. The collected data were entered into SPSS version 27 and AMOS version 24 for further analyses. The researchers used SEM analyses to answer the research questions.

## Results

The researchers used descriptive analysis to find the answer to research question number one. In the following tables, the results of these analyses are presented.

The results of Table 1 show that EFL language teachers accepted the role of technology in language classrooms.

**Table 1:** Frequency and percent of the questions related to EFL teachers’ attitudes to digital literacy.

Strongly agree			Agree		Uncertain		Disagree		Strongly disagree	
	*N*	%	*N*	%	*N*	%	*N*	%	*N*	%
Q1	701	33.2%	1,060	50.2%	258	12.2%	44	2.1%	47	2.2%
Q2	589	27.9%	980	46.4%	426	20.2%	62	2.9%	53	2.5%
Q3	691	32.7%	1,051	49.8%	295	14.0%	33	1.6%	40	1.9%
Q4	630	29.9%	1,058	50.1%	321	15.2%	52	2.5%	49	2.3%
Q5	673	31.9%	1,088	51.6%	274	13.0%	33	1.6%	42	2.0%
Q6	610	28.9%	1,101	52.2%	313	14.8%	41	1.9%	45	2.1%
Q7	582	27.6%	1,028	48.7%	403	19.1%	55	2.6%	42	2.0%
Q8	573	27.2%	1,040	49.3%	403	19.1%	50	2.4%	44	2.1%
Q9	580	27.5%	1,048	49.7%	387	18.3%	53	2.5%	42	2.0%
Q10	566	26.8%	1,020	48.3%	419	19.9%	63	3.0%	42	2.0%
Q11	585	27.7%	1,064	50.4%	374	17.7%	50	2.4%	37	1.8%
Q12	574	27.2%	1,078	51.1%	375	17.8%	46	2.2%	37	1.8%
Q13	607	28.8%	1,113	52.7%	316	15.0%	35	1.7%	39	1.8%
Q14	605	28.7%	1,109	52.6%	317	15.0%	39	1.8%	40	1.9%
Q15	596	28.2%	1,124	53.3%	319	15.1%	28	1.3%	43	2.0%
Q16	584	27.7%	1,072	50.8%	376	17.8%	42	2.0%	36	1.7%
Q17	571	27.1%	1,055	50.0%	390	18.5%	48	2.3%	46	2.2%

The results of Table 2 reveal that nearly 60 percent of EFL language teachers believed that using technology increases students’ participation and engagement in language classrooms (question 26). In addition, more than 55 percent of teachers claimed that technology literacy is required to improve engagement and classroom participation.

**Table 2:** Frequency and percent of the questions related to EFL Teachers’ Attitudes to Digital Engagement.

Never/Very low			Low		Medium		High	
	*N*	%	*N*	%	*N*	%	*N*	%
Q18	280	13.3%	731	34.6%	824	39.1%	275	13.0%
Q19	280	13.3%	719	34.1%	830	39.3%	281	13.3%
Q20	306	14.5%	700	33.2%	827	39.2%	277	13.1%
Q21	272	12.9%	646	30.6%	886	42.0%	306	14.5%
Q22	266	12.6%	599	28.4%	886	42.0%	359	17.0%
Q23	254	12.0%	613	29.1%	903	42.8%	340	16.1%
Q24	273	12.9%	619	29.3%	906	42.9%	312	14.8%
Q25	263	12.5%	630	29.9%	899	42.6%	318	15.1%
Q26	259	12.3%	619	29.3%	911	43.2%	321	15.2%
Q27	259	12.3%	619	29.3%	901	42.7%	331	15.7%

The results of Table 3 represent that more than 60 percent of EFL teachers considered the technological skills of teachers (question 43), the technological mastery of students (question 46), and university infrastructures and facilities (question 38) as the main barriers to conducting technology-integrated language classrooms.

**Table 3:** Frequency and percentage of the questions related to EFL teachers’ attitudes to digital barriers.

A very high barrier			High barrier		Medium barrier		Low barrier		A very low barrier	
	*N*	%	*N*	%	*N*	%	*N*	%	*N*	%
Q28	292	13.8%	551	26.1%	682	32.3%	336	15.9%	249	11.8%
Q29	275	13.0%	564	26.7%	705	33.4%	322	15.3%	244	11.6%
Q30	282	13.4%	555	26.3%	711	33.7%	323	15.3%	239	11.3%
Q31	284	13.5%	589	27.9%	672	31.8%	309	14.6%	256	12.1%
Q32	289	13.7%	560	26.5%	747	35.4%	300	14.2%	214	10.1%
Q33	286	13.6%	586	27.8%	735	34.8%	283	13.4%	220	10.4%
Q34	289	13.7%	554	26.3%	758	35.9%	292	13.8%	217	10.3%
Q35	278	13.2%	588	27.9%	741	35.1%	284	13.5%	219	10.4%
Q36	285	13.5%	575	27.3%	761	36.1%	271	12.8%	218	10.3%
Q37	280	13.3%	601	28.5%	745	35.3%	276	13.1%	208	9.9%
Q38	280	13.3%	586	27.8%	761	36.1%	275	13.0%	208	9.9%
Q39	277	13.1%	587	27.8%	740	35.1%	286	13.6%	220	10.4%
Q40	288	13.6%	565	26.8%	764	36.2%	273	12.9%	220	10.4%
Q41	292	13.8%	617	29.2%	738	35.0%	251	11.9%	212	10.0%
Q42	282	13.4%	577	27.3%	752	35.6%	289	13.7%	210	10.0%
Q43	285	13.5%	571	27.1%	775	36.7%	274	13.0%	205	9.7%
Q44	288	13.6%	578	27.4%	789	37.4%	251	11.9%	204	9.7%
Q45	280	13.3%	623	29.5%	750	35.5%	262	12.4%	195	9.2%
Q46	287	13.6%	575	27.3%	793	37.6%	257	12.2%	198	9.4%

The results of Table 4 show that more than 50 percent of teachers are satisfied with the university’s policy about encouraging the use of tablets and other technologies in their classrooms (question 50).

**Table 4:** Frequency and percent of the questions related to EFL Teachers’ Perceptions of Digital Literacy.

Very Low			Low		Medium		High		Very high	
	*N*	%	*N*	%	*N*	%	*N*	%	*N*	%
Q47	245	11.6%	433	20.5%	690	32.7%	414	19.6%	328	15.5%
Q48	238	11.3%	445	21.1%	700	33.2%	401	19.0%	326	15.5%
Q49	230	10.9%	433	20.5%	705	33.4%	422	20.0%	320	15.2%
Q50	229	10.9%	445	21.1%	706	33.5%	411	19.5%	319	15.1%
Q51	236	11.2%	426	20.2%	693	32.8%	433	20.5%	322	15.3%
Q52	220	10.4%	428	20.3%	715	33.9%	415	19.7%	332	15.7%

**Figure 2:**
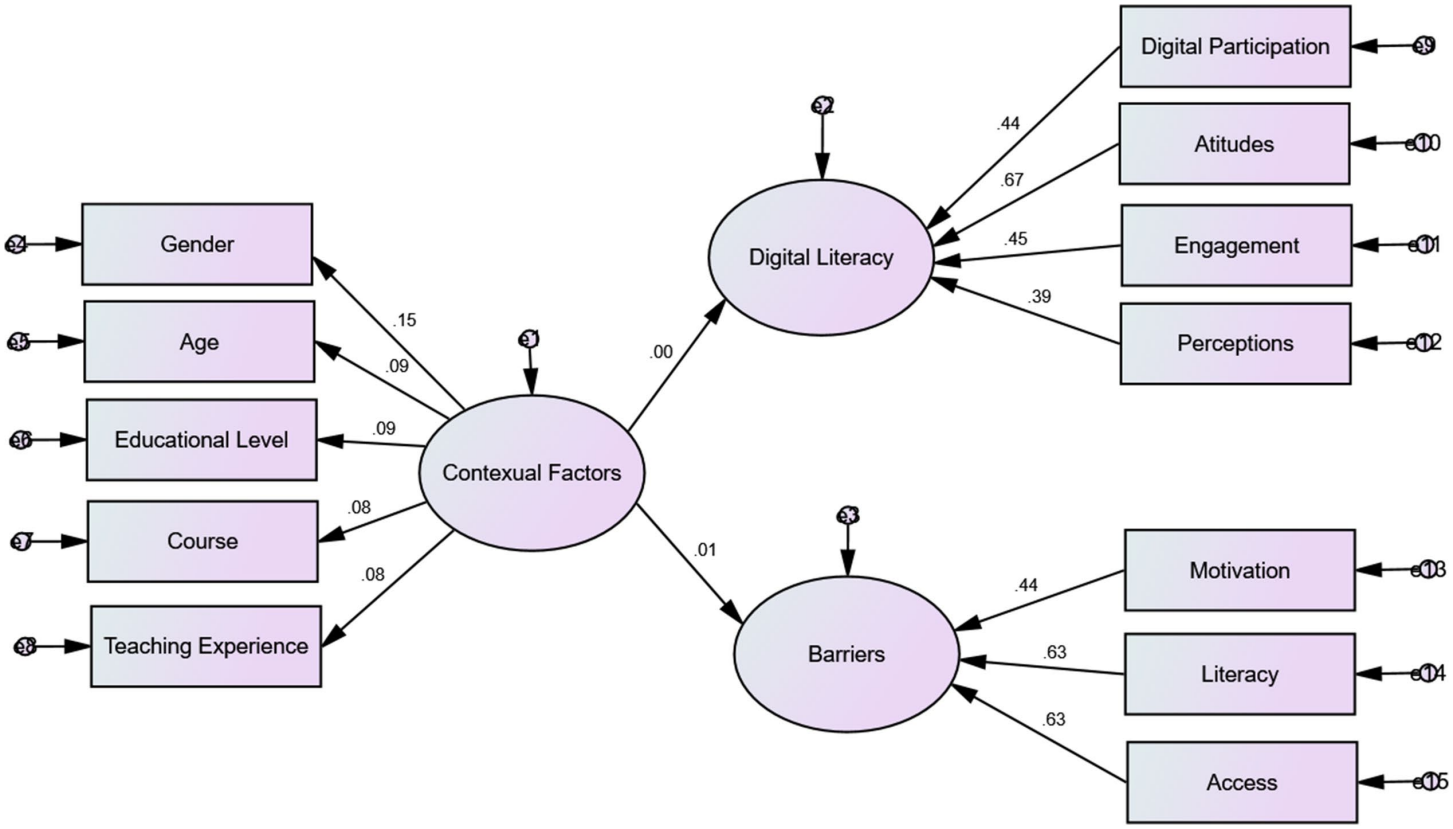
Model fit for the association between contextual factors and digital literacy in the standardized estimation mode.

To answer the second and third research questions, the researchers used SPSS, version 27, and Amos version 24. The results of the analyses are presented in the following Figure 2.

According to the software output, Chi-square = 14930.669, Degrees of freedom = 65, and Probability level = 0.000; Chi-square test is significant (Sig = 0.000 < 0.05), so it can be concluded that there is a significant difference in the frequency of variables. The values of the analyses are presented in the following tables.

As the results of the Tables 5–7 indicated, there was no significant association between contextual factors and digital literacy and barriers to digital literacy.

**Table 5:** CMIN.

Model	NPAR	CMIN	DF	P	CMIN/DF
Default model	25	14930.669	65	0.000	229.703
Saturated model	90	0.000	0		
Independence model	24	14933.156	66	0.000	226.260

**Table 6:** Baseline comparisons.

Model	NFI Delta1	RFI rho1	IFI Delta2	TLI rho2	CFI
Default model	0.000	−0.015	0.000	−0.015	0.000
Saturated model	1.000		1.000		1.000
Independence model	0.000	0.000	0.000	0.000	0.000

**Table 7:** RMSEA.

Model	RMSEA	LO 90	HI 90	PCLOSE
Default model	0.329	0.325	0.334	0.000
Independence model	0.327	0.322	0.331	0.000

However, the analyses showed a significant association between teachers’ digital literacy and their attitudes toward technology, participation in technology-based teaching activities, engagement in technology, and their perceptions of technology in their teaching contexts. In addition, the results show that EFL teachers believed that the major barriers in conducting technology-based language classrooms are teachers’ digital literacy and their access to technology in their teaching contexts.

The results of Tables 8, 9 demonstrated no significant association between teachers’ contextual factors and their digital literacy. However, digital participation predicted about 20 percent of changes in teachers’ digital literacy, their attitudes estimated about 32 percent, their engagement about 26, and their perceptions of technology in their contexts estimated about 19 percent of changes in their digital literacy. In addition, the results showed that teachers’ digital literacy predicted about 40 percent of changes in their attitudes toward barriers to conducting technology-based teaching environments.

**Table 8:** Standardized regression weights.

	Estimate
Age ← Contextual factors	0.093
Level ← Contextual factors	0.094
Course ← Contextual factors	0.084
Teaching experience ← Contextual factors	0.083
Gender ← Contextual factors	0.154
Digital literacy ← Contextual factors	0.004
Barriers to technology integration ← Contextual factors	0.006
Digital literacy ← Digital Participation	0.436
Digital literacy ← Attitudes	0.673
Digital literacy ← Engagement	0.453
Digital literacy ← Perceptions	0.386
Barriers to technology integration ← Motivation	0.443
Barriers to technology integration ← Literacy	0.631
Barriers to technology integration ← Access	0.626

**Table 9:** Intercepts: (Group number 1–Default model).

	Estimate	S.E.	C.R.	*p*	Label
Digital Participation	19.338	0.170	113.611	***	par_6
Attitudes	31.676	0.262	120.743	***	par_7
Engagement	25.830	0.177	146.323	***	par_8
Perceptions	18.466	0.151	122.634	***	par_9
Motivation	13.985	0.116	120.976	***	par_10
Literacy	19.678	0.165	119.465	***	par_11
Access	19.503	0.164	119.263	***	par_12
Gender	1.745	0.010	183.402	***	par_1
Age	1.834	0.016	115.810	***	par_2
Level	1.387	0.016	88.565	***	par_3
Course	1.578	0.017	90.246	***	par_4
TE	1.804	0.018	102.019	***	par_5

## Discussion

This study investigated the digital literacy of English language teachers. In addition, the present study examines the relationship between digital literacy and gender, education level, and teaching experience of English language teachers. The obtained results indicate no significant association between environmental factors and the digital literacy of EFL teachers. It should also be noted that factors such as insufficient training of teachers, and lack of up-to-date textbooks can affect teachers’ digital literacy and put their teaching under the spotlight.

Another finding of the current research was that there was no statistically significant difference between male and female teachers in digital literacy skills. This finding is somewhat consistent with the research findings of Gisbert Cervera and Lázaro Cantabrana (2015), which showed that gender cannot be a strong predictor for the use of information technology and digital tendencies. It can be argued that male and female teachers have equal access to different types of technology and equal opportunities in educational environments, which will lead to the adjustment of the digital divide. Gudmundsdottir and Hatlevik (2018) also found that male and female teachers may have the same access to digital technology. It seems that every teacher is an individual decision-maker in their life and future, and teachers’ digital literacy has more to do with the motivation and will of the person and their cognitive skills and understanding. However, for a better understanding, more research should be done on gender and digital literacy to identify its different dimensions.

Another finding of the current research was that no significant statistical difference was found between teaching experience and digital literacy. Some studies indeed show conflicting results (Ilomäki et al., 2016; Pettersson, 2018; List, 2019; Zimmer et al., 2021), but as the results of this research showed, it seems that digital literacy can be influenced by other factors such as a positive attitude toward technology, knowledge of using technology, access to technology, and context. Similarly, Tawafak et al. (2023) argued that the digital tools and teachers’ literacy skills are different in various contexts and cultures.

Consistent with the literature, this research found that there was no statistically significant difference between the level of teachers’ education and their digital literacy (Tondeur et al., 2017; Katia and José Tejada, 2018; Fu and Wang, 2022; Palacios-Hidalgo and Huertas-Abril, 2022). One of the reasons for that is the need to update the digital knowledge of teachers with doctorate degrees and the need for them to be constantly aware of educational and social contexts. Educational environments do not have specific goals, programs, and policies regarding teachers’ digital literacy. In other words, the learning and application of digital literacy can be related to the targeted and compulsory policies of educational environments; the responsibility of teachers in the use of digital tools, and the training of teachers through digital literacy workshops. These results may be explained by the fact that the environment, conditions of learning, teaching, and life of teachers may be different, and they may not have enough access, support, and training before, during, and after the beginning of their career. The study demonstrated a strong association between access to technology and teachers’ digital literacy. However, an adequate interaction between teachers and students are among the major challenges of learning environments that teachers or students do not have the required digital knowledge. The finding is in consistent with what Pikhart et al. (2022) argued. These relationships may partly be explained by two important factors. On the one hand, digital literacy can be deeply affected by the lack of digital technology facilities in educational environments. On the other hand, the education and training of teachers and the compilation of textbooks are not following the modern methods of learning and teaching English. As a result, the literacy of teachers is marginalized in some dimensions, including cognitive.

## Conclusion

Although the results of this study showed no significant relationship between environmental factors such as age, gender, education, and their digital literacy, factors such as teachers’ attitudes, their skills in using technology, and their access to technology have a significant impact on their digital literacy. This important result can be rooted in access to digital resources and their use in society. More access to digital technology can increase the digital literacy of language teachers, and digital literacy can make language teachers more capable of teaching and optimal use of digital technology in the digital age in a way that facilitates the teaching process and as an available tool should be used for educational purposes. Therefore, the need to attract, empower, and retrain teachers before and during service should be given serious attention.

The rapid growth of human knowledge resources and the emergence and expansion of information and communication technologies have made mankind today face unprecedented confusion in finding information sources. The only way to overcome this confusion and speed up finding the required information is to increase digital literacy skills. Digital literacy skills have been considered due to the expansion of communication networks and electronic resources and the selection of the best and most relevant source. A literate person in terms of information technology can use computers, application software, databases, and other technologies to perform various tasks related to his education, career, and personal affairs; Therefore, people who want to achieve information literacy must first acquire the relevant technological skills.

According to the findings of this research, some practical suggestions can be made to the policy makers, planners and implementers of the education and training system:Measuring the level of computer literacy of teachers should be considered as a basis for their evaluation; By holding educational workshops and in-service training courses and by adding the topic of classroom management to the subject of teacher training centers and teacher training courses, actions should be taken to improve the behavior style of teachers in classroom management; special attention should be paid to new teachers, in terms of the need to understand digital literacy, receive sufficient training and feel responsible for their own education; in order to improve educational quality and coordinate with digitally literate teachers, students should also receive the necessary training for the optimal use of technology; the authors of textbooks should compile books with new content and materials to be in harmony with new environments, and policy makers should also try to make fundamental changes in English language education programs. Learning from the findings of this study, when planning or delivering technology-based learning environments, teachers need to first understand the students in terms of their level of digital literacy for learning. A good fit of digital literacy level to course expectations is necessary for successful technology-based learning. If some students have low digital literacy levels, additional exercises, and tutorials can be used to help these students improve their digital literacy capabilities. Carolus et al. (2023) support that students can learn to use educational technologies not familiar to them for learning if they are introduced to and given a chance to use these technologies. Students might require a little time and instruction for them to arrive at the mark of digital literacy. Before teachers coordinate new technology into their illustrations/educational program or energize digital progressions/literacy, they need to survey what digital devices their students know all about and how these apparatuses can be utilized in the classroom. Advancing digital literacy in the classroom prompts students to think about learning and improvement and look at their demeanor toward technology. All the more explicitly, through this instructive cycle, students foster methods where they can equitably consider their learning, progress, and endeavors. This could include taking part in technology courses or consolidating really learning technology in everyday subject conveyance. Teachers can assist their students with building an intelligent practice by empowering them to record their contemplations about what they have realized and how they have taken care of the utilization of digital devices.

The participants in this study had different technology usage experiences. How familiar they were with technology could influence their views of its usefulness was in their teaching process. In addition, the participants were from different courses and provinces. The types and levels of digital literacy capabilities required could be diverse across subject matter areas. The study context was a local university that had adopted blended learning. Future studies can examine the original research model in different contexts, for instance, primary or secondary schools, professional courses, working adults, and full-time young students, etc. Future studies can consider new factors, investigate interactions among the factors, and introduce moderators. More studies are required to generalize the findings of the present study. Future studies can focus on the quality of teacher training courses and investigate their adequacy in longitudinal studies. Teachers’ beliefs in different contexts can be investigated in EFL contexts. Learners’ beliefs and their digital literacy can be scrutinized in future studies. Digital literacy abilities offer colossal advantages and benefits to the two teachers and students, nonetheless, without legitimate use and understanding, the use of digital apparatuses can become overpowering, or even perilous, particularly with regards to more youthful age gatherings. Youngsters right now live in a developing digital world that requires expanding capacities and abilities to utilize and adjust digital apparatuses. While there is understanding that another arrangement of abilities including innovations is imperative for the instructive improvement of students, there is little agreement about definitively what information and capacities are important for youngsters to be viewed as digitally educated. Future studies could investigate young learners’ understandings of digital literacy and the specific skills they require to cope with this new mode of instruction.

## Data availability statement

The original contributions presented in the study are included in the article/supplementary material, further inquiries can be directed to the corresponding author.

## Ethics statement

The studies involving human participants were reviewed and approved by Jiaozuo Normal College Academic Ethics Committee. The patients/participants provided their written informed consent to participate in this study.

## Author contributions

The author confirms being the sole contributor of this work and has approved it for publication.

## Conflict of interest

The author declares that the research was conducted in the absence of any commercial or financial relationships that could be construed as a potential conflict of interest.

## Publisher’s note

All claims expressed in this article are solely those of the authors and do not necessarily represent those of their affiliated organizations, or those of the publisher, the editors and the reviewers. Any product that may be evaluated in this article, or claim that may be made by its manufacturer, is not guaranteed or endorsed by the publisher.
